# Grape Canes (*Vitis vinifera* L.) Applications on Packaging and Particleboard Industry: New Bioadhesive Based on Grape Extracts and Citric Acid

**DOI:** 10.3390/polym14061137

**Published:** 2022-03-12

**Authors:** Jorge Santos, João Pereira, Danilo Escobar-Avello, Irene Ferreira, Carlos Vieira, Fernão D. Magalhães, Jorge Manuel Martins, Luísa H. Carvalho

**Affiliations:** 1 ARCP—Associação Rede de Competência em Polímeros, 4200-355 Porto, Portugal; jamp@fe.up.pt; 2 LEPABE—Faculty of Engineering, University of Porto, Rua Dr. Roberto Frias, s/n, 4200-465 Porto, Portugal; fdmagalh@fe.up.pt (F.D.M.); jmmartins@estgv.ipv.pt (J.M.M.); lhcarvalho@estgv.ipv.pt (L.H.C.); 3 Unidad de Desarrollo Tecnológico, Universidad de Concepción, Coronel 4191996, Chile; daniescobar01@gmail.com; 4 Centro Nacional de Excelencia para la Industria de la Madera (CENAMAD), Pontificia Universidad Católica de Chile, Av. Vicuña Mackena 4860, Santiago 7820436, Chile; 5 FWFI—Freshwood Forms Industry, 2430-600 Vieira de Leiria, Portugal; irene.ferreira@freshwood.eu (I.F.); carlos.vieira@freshwood.eu (C.V.); 6 DEMad—Department of Wood Engineering, Instituto Politécnico de Viseu, Campus Politécnico de Repeses, 3504-510 Viseu, Portugal

**Keywords:** vine shoots, biomass, valorisation, polyphenols, packaging, particleboard, citric acid, FTIR-ATR

## Abstract

The main by-product generated in the wine industry are the grape canes, derived from the pruning process. In order to increase the valorisation possibilities of this highly polyphenolic lignocellulosic material, this work focuses on its applicability in the materials industry. As a first step, we demonstrate the viability of using grape cane particles as raw material for particleboard production, combined with a melamine formaldehyde urea (MFU) binder. In addition, looking for the application of these particleboards in the food packaging industry, particleboards based on grape canes were also produced using a new bioadhesive, obtained from the grape cane extract and citric acid. The self-condensation reaction of the grape cane extracts, and the curing reaction with citric acid, were studied by FTIR-ATR and ABES showing the feasibility of this new bioadhesive formulation. Looking for a zero-waste process, the effect of the type of raw material (fresh grape cane, solid by-product of the extraction) and of the extract used on the properties of particleboard were also studied. Citric acid was demonstrated to be a good crosslinking agent for grape cane extract. This work shows that it is possible to produce a new lignocellulosic product based only on grape cane particles using a binder based on grape cane extracts and citric acid. The implemented methodology allowed producing particleboards with applicability in the food-packaging industry, minimizing the waste generated in the process.

## 1. Introduction

Grape canes, also called vine-shoots, are the most important by-products in viticulture, although other by-products such as grape seeds, pomace, stalks and skins are also generated in the vinification process. All of them could be a low-cost raw material for the production of high value-added phytochemicals mainly by its polyphenolics compounds profile [1,2] to be used as food additives or in the pharmaceutical industry. Each year, around 14.8–29.6 million tons of grape canes are produced globally [3]. Grape cane valorisation is not a new topic, and its potential to obtain extracts with high polyphenolic content was studied by other authors [1,2,4]. Other chemical components of grape canes are carbohydrates (27.5%), lignins (38.7%), proteins (6.7%), and minerals (1%) [2]. The characterization of the extracts obtained from different varieties of grape cane has shown the presence of a high amount of stilbenes [5,6] and condensed polyphenols. The valorisation of this lignocellulosic material was focused on the production of extracts for its application on the pharmaceutical and food industry, due to which most of the studies were performed with alcoholic solvents [2,6,7]. However, to our knowledge, no research work has been carried out focusing the application of grape cane extract in the materials industry. Unlike the pharmaceutical industry, the application of grape cane extracts in the formulation of new lignocellulosic materials makes it necessary to study extraction methodologies with low economic cost and low environmental impact.

Until now, the valorisation of grape canes as lignocellulosic material has focused on its use as a raw material for particleboards production, using traditional fossil-based adhesives as binders [8,9,10,11]. In this work, following the same procedure previously carried out by this research group in the valorization of pine by-products [12], the viability of using grape cane particles as raw material in the production of particleboards was studied. For this, a melamine formaldehyde urea (MFU) resin, catalyzed with citric acid, was used as a binder. The viability of citric acid to be used as a catalyst for MFU resin, and its influence on the curing reaction mechanism, was previously studied by this research group [13].

Considering the use of these particleboards in the food packaging industry, it is interesting to look for new bioadhesives with less toxicity and environmental impact than those traditionally used in the particleboard industry for furniture applications. In particular, considering the packaging of products from the wine industry, the grape cane by-product could be used as source of the particles and of the bioadhesive for the particleboard production. A similar approach was followed before by this research group in the valorisation of cardoon by-products from the cheese industry [14]. The first step of the study was the extraction process, which was carried out using the alkaline extraction methodology [15,16]. The influence of the alkaline extraction agent (NaOH, NaHCO_3_) used in the extraction process was analysed. Looking to improve the sustainability of the process and achieve the objective of a zero-waste process, the influence of the extraction treatment on the grape cane particles was studied and the solid by-product generated was characterised by FTIR-ATR. The feasibility of using the solid fraction obtained after the extraction process as raw material in particleboard production was also evaluated. The influence of the raw material (fresh grape cane, solid by-product of the extraction with NaOH and the solid by-product from the extraction with NaHCO_3_) and the extract used in the formulation of the bioadhesive on the properties of particleboard were assessed.

The grape cane extracts self-condensation reaction, and the curing reaction with citric acid was studied by FTIR-ATR and ABES. The solid by-product obtained after the extraction process was dried and used as raw material to produce new particleboards. The influence of the extraction conditions on the material was also evaluated.

The aim of this study was to show that grape cane particles may be used as a raw material in particleboard manufacture, using a bioadhesive formulated with grape cane extract and citric acid as binder. For this, the research methodology followed was: to first study the feasibility of using grape cane particles as raw material in the production of particle board, then to evaluate the grape cane extraction process and its influence on the properties of the extract, and finally to study the behaviour of the extracts as a component in the formulation of a bioadhesive with citric acid.

For the first time, a particleboard based only in grape cane and citric acid was produced, and its applicability in the packaging industry demonstrated.

## 2. Materials and Methods

### 2.1. Raw Material

Grape canes (*Vitis vinifera* L.) from the Souson (also known as Sousão in Portuguese) variety were collected from healthy plants in an organic vineyard of the D.O. Rias Baixas at Vila de Cruces (Galicia, NW Spain) (42°47′ 32.9′′ N 8°15′09.7′′ W at 827 m of altitude).

Grape canes (GC) were grounded using a 4 mm filter in a cutting mill (Retsch, Haan, Germany). The GC was then sieved using a vibrating sieve shaker (Retsch, Haan, Germany) to select the proportion of particle size between 1 and 4 mm. The materials were then oven-dried at 60 °C until they reached the desired moisture content. Euroresinas, Indústrias Químicas, S.A. (Sines, Portugal) offered formaldehyde (37 wt % solution), melamine, sodium hydroxide (10 wt % solution), and urea. Citric acid was provided by PanReac AppliChem (Barcelona, Spain).

### 2.2. Extraction

Solid/liquid ratio was fixed at 1/10 (*w*/*w*) for all the extraction experiments. Aqueous solutions of sodium carbonate or sodium hydroxide were used, with an extraction temperature of 90 °C. The materials were mixed with water at room temperature, heated, and, once the temperature of 90 °C was attained, sodium carbonate or sodium hydroxide solutions were added. After 1 h of extraction, the suspension was vacuum filtered, the solid residue was washed under water flow, and the aqueous extract solutions were concentrated in a rotary evaporator (BÜCHI, Flawil, Switzerland). The extraction experiments and the product analysis (FTIR, ABES, extraction yield) were carried out in triplicate and the results averaged. Table 1 shows the extraction conditions used.

The extraction yield was calculated by measuring the difference between the initial dry material weight and the ends waste dry material weight (Equation (1)):

(1) $$EY\left(\%\right)=\frac{Raw\;material\left(g\right)-Waste\;material\left(g\right)}{Raw\;material\left(g\right)}\times 100$$

### 2.3. Fourier Transform Infrared Spectroscopy (FTIR) Assay

FTIR spectra were acquired using a VERTEX 70 FTIR spectrometer (Bruker, Billerica, MA, USA) with a high sensitivity DLaTGS detector. All the tests were done at room temperature. ATR mode was used to evaluate all the samples. An A225/Q PLATINUM ATR Diamond crystal with a single reflection accessory was used. The spectra were obtained with a resolution of 4 cm^−1^ from 4000 to 400 cm^−1^. OPUS 7.0 software was used to record and analyze all spectra.

### 2.4. Automated Bonding Evaluation System (ABES) Assay

The maximal strength of the wood-adhesive-wood system was evaluated using an ABES instrument (Adhesive Evaluation Systems, Corvallis, OR, USA) at set temperature and time conditions [17].

Before testing, the wood veneer samples (*Fagus sylvatica* L., thickness 0.7 mm) were stored in a conditioned environment for one week at 20 °C and 53% relative humidity. Probes were cut into 117 mm × 20 mm strips using a pneumatically-driven sample cutting device for ABES sample preparation (supplied by Adhesive Evaluation Systems, Corvallis, OR, USA).

Two peeled wood veneer strips were glued along the fiber direction with a 100 mm^2^ overlap. For all the tests, 20 mg of adhesive was used. Analyses were performed at 120 and 160 °C after pressing times of 30, 60, 90, 120, 150, and 180 s. Measurements were done five times for each data point.

### 2.5. Particleboard Manufacture and Testing

Two sets of particleboards were prepared. The first set was one layer 210 mm × 210 mm × 16 mm particleboards at a specific pressure of 4 MPa and 190 °C of press temperature for 3 min.

A second set of panels was prepared at the same pressing and temperature conditions, but in this case, 210 mm × 210 mm × 8 mm for 10 min.

The first set of particleboards was produced with grape canes and using as reference a standard industrial-grade mix of wood particles (30% maritime pine, 15% eucalypt, 25% pine sawdust and 30% recycled wood). Particles were blended with a synthesized MFU resin (10% mass of resin solids/mass of dry wood), paraffin (3% wt) and catalyst (citric acid (3% wt) in a laboratory glue blender. MFU resin was synthesized following the procedure described in previous works by the group [12,13].

The second set of particleboards was produced also with grape cane particles but using a bioadhesive based on the grape cane extract (GC_E_) and citric acid (10% mass of resin solids/mass of dry wood) as binder.

In the first set of panels, the glued mat was pressed to produce a board with a target density of 650–670 kg m^−3^ and 500–550 kg m^−3^ in the second set of panels.

Particleboards were subjected to physical and mechanical testing in accordance with European standards, such as density (EN 323: 1993), moisture content (EN 322: 1993), thickness swelling (EN 317: 1993), water absorption (EN 317: 1993) and internal bond strength (EN 319: 1993). The original board samples were tested for internal bond (IB) strength. The classification of the boards was done in accordance with EN 312: 2010.

## 3. Results and Discussion

Dry grape canes were extracted using an alkali extraction methodology, to obtain an extract solution with potential applicability in the bioadhesive formulation. Thinking of a future industrial application and from an economic point of view, the viability of the process will be conditioned by the extraction yield of the process.

Table 2 shows the extraction agents’ influence on the extraction yield obtained.

Regarding the values obtained, the higher values of extraction yield were achieved when sodium hydroxide was used as an extraction agent. The extraction yield values obtained were similar to those obtained for other lignocellulosic by-products with high applicability in bioadhesive formulation [12,14,18,19].

### 3.1. FTIR-ATR Characterization

For an integral valorisation of the lignocellulosic by-products generated in an industrial process, it is necessary to characterize all of them and find their best industrial application.

In this sense, the present study analysed the grapes canes without any treatment (GC), the extraction residue (GC_ER_) and the alkali grape canes extract (GC_E_).

**Figure 1:**
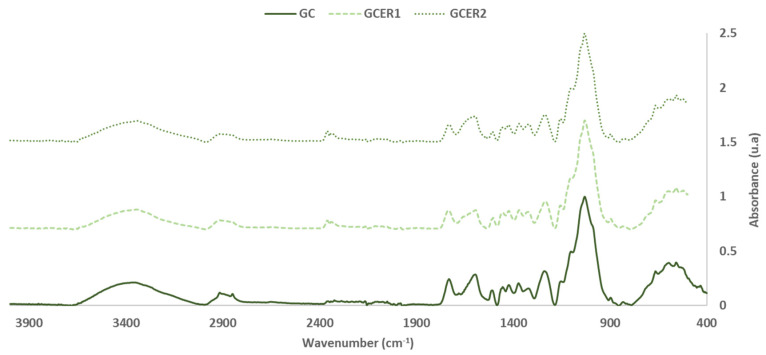
FTIR spectra: Solid grape canes before (GC) and after (GC_ER1_, GC_ER2_) the extraction process (400–4000 cm^−1^).

**Figure 2:**
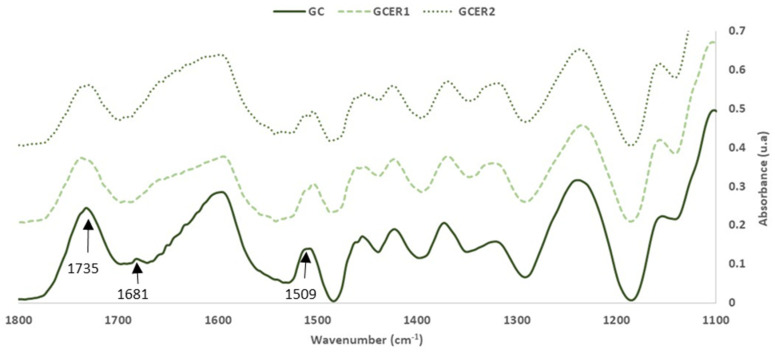
FTIR spectra: Solid grape canes before (GC) and after (GC_ER1_, GC_ER2_) the extraction process (1100–1800 cm^−1^).

FTIR-ATR spectra of GC, GC_ER1_, GC_ER2_ (the extraction residue for the extract 1 and for the extract 2, respectively) are shown in Figure 1 and Figure 2.

Based on the intensity of the vibration bands in the GC and GC_ER1_ and GC_ER2_ spectrum, and as expected, the most abundant compounds in both materials were cellulose, hemicelluloses and lignin. However, bands from other low molecular weight sugars and polyphenolic compounds are also detected. Table 3 summarizes the FTIR band assignments based on previous studies [15,20,21,22,23,24]. For the analysis, the FTIR spectra were normalized based on the most intense band of the C-O, C-C, and C-C-O stretching vibrations of cellulose, hemicelluloses and lignin at 1025–1035 cm^−1^ [22].

As expected, the main differences detected, due to the extraction process, were observed on the intensity of the bands, due to the extractable compounds present in the material. Regardless of the extraction agent used in the extraction process, the bands assigned to the polyphenolic compounds present in the material, at 1070, 1500 and 1680 cm^−1^, decrease their intensity or disappear in the spectra of the solid fraction obtained after the process extraction.

The characteristic FTIR bands of hemicelluloses and cellulose are very close [25]. The band around 1735 cm^−1^ is due to the C=O stretching vibration of carboxyl and acetyl groups characteristic of hemicelluloses presence. Regarding the intensity of this band, it is possible to evaluate the reduction of the hemicelluloses content in the extracted materials.

In addition, the reduction in the intensity of the bands due to the presence of -CH_2_- groups that appears around 2920 and 2850 cm^−1^ is also noticeable. This effect is associated with two possible processes that could occur in the extraction process, the carryover of fats and oils present in the original material during the extraction process, or the oxidation, or the hydrolysis of CH_2_ groups present in compounds that have not been extracted such as lignin.

In relation to the application studied, the reduction in the intensity of this band is related to the decrease in the hydrophobicity properties of the material, and influences the wettability of the material by the water-based adhesive, and the water-resistance properties of the particleboard produced with the material [12,13].

**Figure 3:**
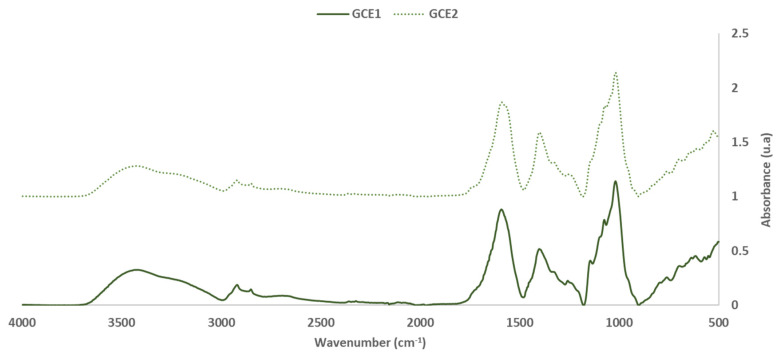
FTIR spectra: Grape cane extracts GC_E1_, GC_E2_ (400–4000 cm^−1^).

**Figure 4:**
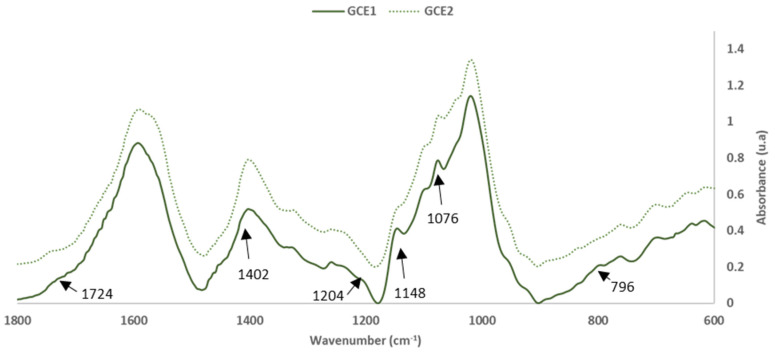
FTIR spectra: Grape cane extracts GC_E1_, GC_E2_ (600–1800 cm^−1^).

To evaluate the extractable components, FTIR-ATR of the oven-dried (60 °C) extracts GC_E1_ and GC_E2_ (Extracts 1 and 2) analysis was done, and the spectrum obtained is shown in Figure 3 and Figure 4.

Table 4 shows the characterization of the mains peaks of the two extracts (GC_E1_ and GC_E2_) in which the intensity values were normalized with the aromatic polyphenol ring vibration at 1600 cm^−1^. The band assignment was performed based on previous studies [15,20,21,22,23,24].

The high intensity of the main bands, associated with the presence of condensed tannins at 1454, 1204 and 1020 cm^−1^ in the GC_E1_ spectra versus the GC_E2_ spectra, confirms the higher content of these compounds in the extract obtained using NaOH as the extraction agent. Otherwise, the bands due to the presence of stilbene compounds in the extract were similar or slightly higher for the extract obtained with NaHCO_3_ as the extraction agent.

To conclude the FTIR-ATR study of both extracts, the spectral bands related to the presence of sugars and carbohydrates (from hemicellulose) in both extracts (1720–1740, 1145 and 800 cm^−1^) were analysed. The study showed that there was a higher percentage of these compounds in the extract obtained with NaOH than in the extract obtained with NaHCO_3_.

### 3.2. Extracts Reactivity

#### 3.2.1. FTIR-ATR Results

**Figure 5:**
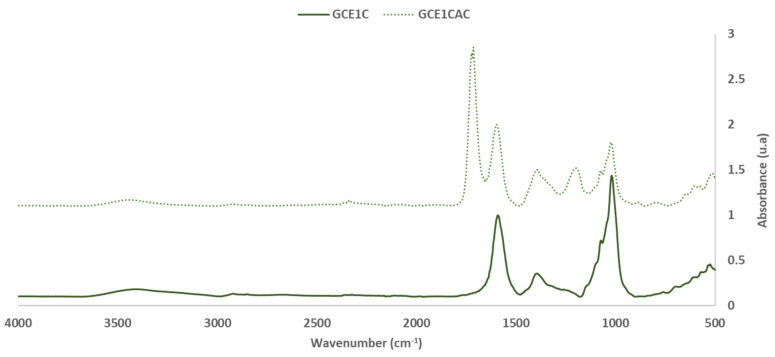
FTIR spectra: Grape cane extracts GC_E1_, cured alone GC_E1_C, and with citric acid GC_E1_CAC (400–4000 cm^−1^).

**Figure 6:**
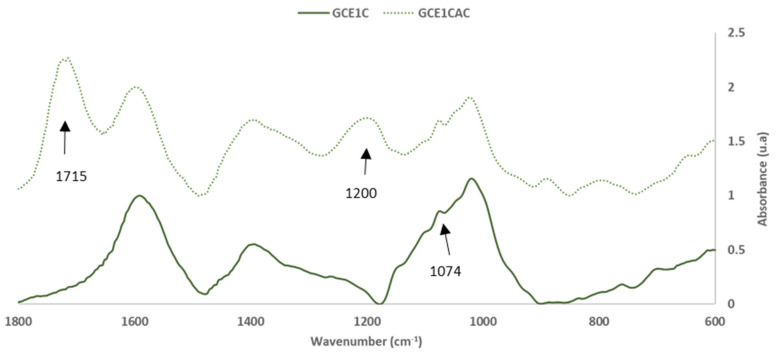
FTIR spectra: Grape cane extracts GC_E1_ cured alone GC_E1_C, and with citric acid GC_E1_CAC (600–1800 cm^−1^).

The FTIR-ATR technique was used to evaluate the chemical reactivity of grape cane extracts GC_E1_. For this, the extract solution (30.0 ± 0.5% of solid content), alone and with 33% (on adhesive weight, dry basis) of citric acid (CA), were reacted at 120 °C for 120 s. The objective was to evaluate the chemical reactivity of the extract, alone (self-condensation) and with a citric acid crosslinker. Figure 5 and Figure 6 show the FTIR-ATR spectra for the extracts cured alone and with citric acid.

For the analysis, the FTIR spectra were normalized, based on the aromatic polyphenol ring vibration band at 1600 cm^−1^ [12].

Table 5 shows the characterization of the mains peaks of the GC_E1_ extract cured alone and with citric acid. The band assignment was performed based on previous studies [15,20,21,22,23,24].

The intensity of the broad band at 3400–3300 cm^−1^ due to the OH stretching vibrations decreases in the curing reaction because hydroxyl groups are involved in the condensation reaction of polyphenols. The intensity of this band in the spectra of the cured GC_EC1_ extract alone and with citric acid was the first signal that confirms the influence of citric acid in the curing reaction.

The band at 1715 cm^−1^, which is related to the axial stretching of the carbonyl groups (C=O) in the carboxylic acid groups, and the band at 1200 cm^−1^ due to the stretching vibration of the CO bonds of the -O-(C=O)- groups (showing the formation of methylene ester bonds [30]) in the GC_EC1+_ CA spectrum also confirms the citric acid participation on the curing reaction.

In addition, the intensity of the band at 1074 cm^−1^ of the condensed tannins, due to the C-O primary alcohol stretching vibration, decreases in the spectra of GC_E1_ with citric acid, indicating the reduction in the percentage of -COH primary alcohol due to the increase in the crosslinking between polyphenolic or sugars units.

##### Influence of Temperature in the Curing Reaction of GC_E_ with Citric Acid

Once the citric acid reaction with the grape cane extract was demonstrated, the influence of the temperature and citric acid percentage used in the bioadhesive formulation was also studied. For that, the GC_E1_ was mixed first with citric acid (33% (on adhesive weight, dry basis)) and reacted at 100, 120, 140, 160 and 180 °C for 120 min.

**Figure 7:**
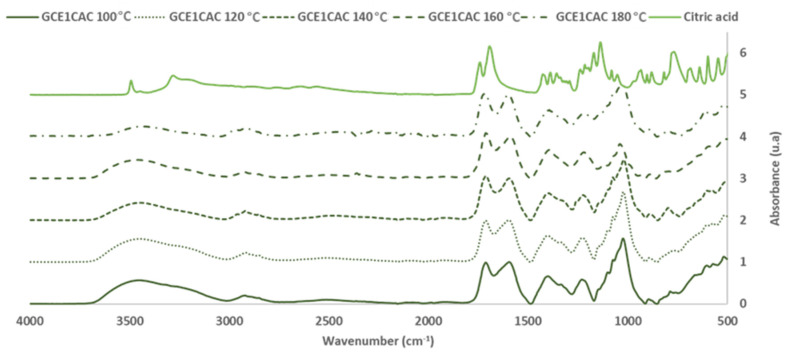
FTIR spectra: Citric acid and Grape cane extract GC_E1_ cured with citric acid (33% on adhesive weight, dry basis) at 100–180 ° C (400–4000 cm^−1^).

**Figure 8:**
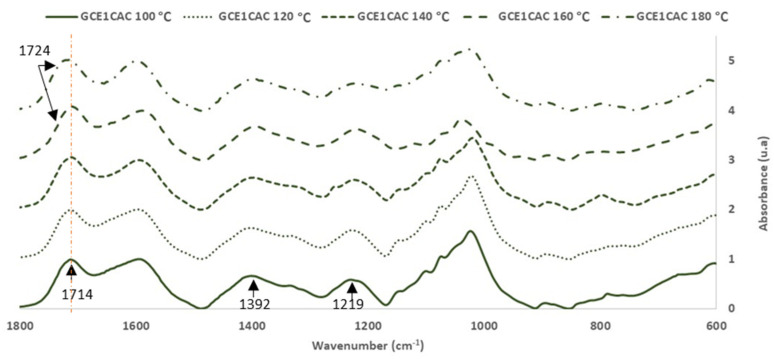
FTIR spectra: Grape cane extract GC_E1_ cured with citric acid (33% on adhesive weight, dry basis) at 100–180 °C (600–1800 cm^−1^).

Figure 7 and Figure 8 show the FTIR-ATR spectra for the extracts cured alone and with citric acid.

For the analysis, the FTIR spectra were normalized based on the aromatic polyphenol ring vibration band at 1600 cm^−1^.

Regarding the most sensitive FTIR-ATR bands involved in the curing reaction, it was possible to evaluate the influence of the temperature on the curing reaction.

The changes of the main bands in the polymerization process are shown in Table 6.

The broad band (3000–3700 cm^−1^) is associated with the OH stretching of the methylol group of polyphenol and the unreactive OH from polyphenols. The methylol condensation (-CH_2_OH) with the free active sites of other phenolic rings is usually the most critical reaction in a polyphenolic adhesive curing process. As the glue achieves a high polymerization degree, the relative intensity of this band decreases [24]. In the phenolic groups, the deformation vibration of the C-C bonds is absorbed in the range of 1400–1500 cm^−1^. Due to system volume constriction, this peak exhibits a progressive reduction as polymerization occurred [24,30]. The band around 1200 cm^−1^ is associated with the C-O stretching vibrations of the polyphenols. The intensity of the band decreases due to the polymerization process. The intensity of the bands at 2920 and 2850 cm^−1^ due to the stretch vibration modes of the CH_2_ groups increases with the polymerization process due to the production of methylene bridges. The changes produced in these characteristic bands of the polyphenolic reaction are a good indicator of the degree of polymerization reached for each curing temperature.

Furthermore, the interaction between citric acid and GC_E1_ was demonstrated by the variations produced in the bands of the C=O groups (due to citric acid). This band, originally at 1738 cm^−1^ in the citric acid spectrum, appears at 1714 cm^−1^ in the spectra of adhesives cured at low temperature (100–140 °C). However, in the spectra of the bioadhesives cured at high temperature (160–180 °C), a new overlapping band appears at higher wavenumber values (1724 cm^−1^) (due to the methylene ester bonds formation). Table 6 also shows that the area of the corresponding bands increases with the reaction temperature.

Regarding the area of the bands of the main peaks involved in the curing reaction of CG_E1_ with citric acid (Table 6), it is seen that increasing the reaction temperature increases the degree of crosslinking of the adhesive achieved, until reaching a temperature of 160 °C.

**Figure 9:**
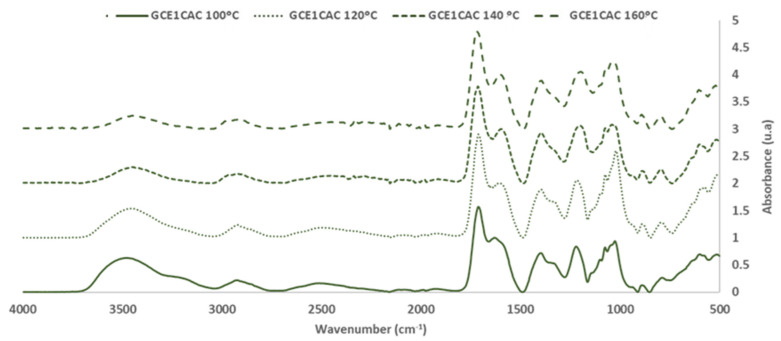
FTIR spectra: Citric acid and Grape cane extract GC_E1_ cured with citric acid (43% on adhesive weight, dry basis) at 100–180 °C (400–4000 cm^−1^).

**Figure 10:**
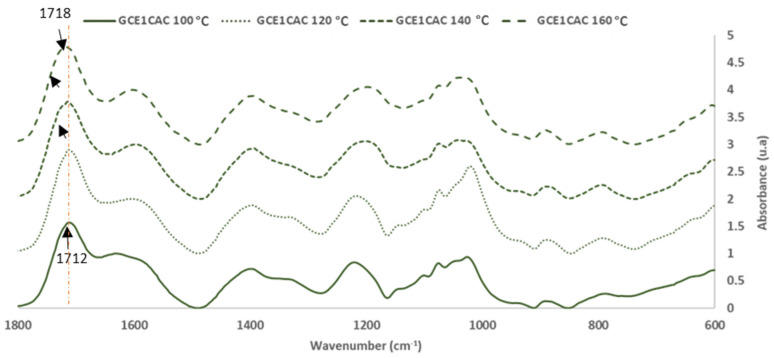
FTIR spectra: Citric acid and Grape cane extract GCE1 cured with citric acid (43% on adhesive weight, dry basis) at 100–180 °C (600–1800 cm^−1^).

To complete the study of the reaction between GC_E_ and citric acid, the influence of increasing the percentage of the latter on the curing reaction was studied. The bioadhesive was formulated mixing CG_E1_ with citric acid to obtain a percentage of 43% on adhesive weight (dry basis). Then, the bioadhesive was cured for 120 s at 100, 120, 140 and 160 °C. Figure 9 and Figure 10 showed the FTIR-ATR spectra of the cured product.

The changes of the main bands in the polymerization process are shown in Table 7.

Regarding the decrease of the band areas of the spectra peaks involved in the curing reaction (Table 7), it is seen that increasing the amount of citric acid in the formulation leads to a decrease on the temperature necessary to produce the reaction with the polyphenolic components of GC_E1_. With 33% CA, it was necessary 160 °C to start the reaction, while, when increasing the percentage of CA to 43%, a significant reduction of the band areas occurs at 140 °C.

#### 3.2.2. ABES Results

**Figure 11:**
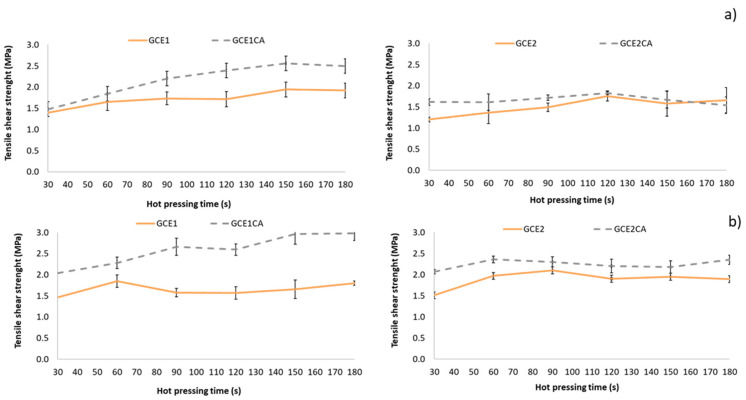
Development of shear strength as a function of hot-pressing time of GC_E1_ and GC_E2_ solutions alone and with citric acid (33 (% on adhesive weight, dry basis)) at (**a**) 120 °C and (**b**) 160 °C.

ABES equipment was used to evaluate the curing of an adhesive in terms of the shear strength of wood-adhesive junctions. This method allows for evaluating how the bond strength develops under controlled hot-pressing conditions. At a constant temperature of 120 °C, different press times (15–120 s) were tested (Figure 11). The adhesive behaviour was evaluated for the aqueous solutions of the two extracts alone (self-condensation reaction), and mixed with citric acid (33% on adhesive weight, dry basis).

Regardless of the extraction conditions used, the ABES evaluation of the mechanical cure reaction confirms the results obtained by the FTIR-ATR evaluation (chemical cure), in which it was demonstrated that citric acid could be used as a biohardener mixed with the Grape cane extracts in the formulation of bioadhesives.

The results also show that the GC_E1_ obtained using NaOH as an extraction agent presents a slightly better mechanical performance as a bioadhesive, when mixed with citric acid, than the extract obtained using NaHCO_3_ (at the studied conditions).

### 3.3. Particleboard Performance

The potential of grape cane to be used as a wood substitute in particleboard production was evaluated combining the non-treated grape canes, and the extraction residue with a MFU resin catalysed with citric acid. The particleboard production methodology was previously reported for the valorisation of non-timber by-products from maritime pine [12].

Table 8 shows the influence of the by-product on the properties of the particleboard.

The behaviour of the particleboards, obtained using grape cane particles as a substitute of the usual industrial wood mix particles, shows its viability to be incorporated as raw material in the production of industrial particleboards. The particleboards obtained from “fresh” grape canes particles, or even from the solid by-product obtained after the solid/liquid extraction, comply with the IB dry resistance standard of the European standard EN 312 for boards type P2, for interior fitments (including furniture) for use in dry conditions (≥0.35 MPa). The physicochemical properties of the particleboards produced using these viticulture by-products were actually better than the obtained with a standard mix of wood particles.

The removal of the extractable fraction had a slightly positive influence on the internal bonding of the particleboards (results for GC_ER1_); however, the water resistance was reduced. As was seen in the FTIR-ATR analysis (Figure 3 and Figure 4), the extraction process treatment causes a decrease in hydrophobicity, which could justify both better adhesive wettability and lower water-resistance.

The use of grape canes as wood-substitute has the advantage of reducing the carbon footprint of the end product, due to the use of a fast-growing crop [14] that is a by-product of the necessary pruning process.

Next, the formulated bioadhesive based on grape cane extract (GC_E_) and citric acid was tested as a binder for the grape cane particleboards.

Two sets of particleboards were produced. One used “fresh” milled grape canes, with the two bioadhesives formulated with grape cane extract and citric acid (GC_E1_ and GC_E2_). The other used the “treated” particles (extraction process by-product) mixed with the same two bioadhesives.

Table 9 and Table 10 show the influence of the extraction conditions used on the properties of the particleboards.

The internal bond and bending strength values obtained for the 100% biobased particleboards do not comply with the requirements of CEN/TS 16368 Lightweight Particleboards—Specifications for general purpose lightweight boards for use in dry conditions (Type LP1). However, the results were similar to those obtained by other authors for particleboards produced using insect rearing residue and rice husks with starch/citric acid mixture as a natural binder [31], rice husks using soy protein-derived bio-binder [32], and with cardoon stalks using cardoon leaf extract and citric acid as bio-adhesive [14].

A considerable decrease in bending strength is observed when the “treated” particles (GC_ER1_ and GC_ER2_) were used as raw material in the particleboard production, regardless of the extraction agent and of the bioadhesive used.

The extraction conditions influence the water resistance of the particleboards. When NaOH used as extraction agent (GC_E1_), the particleboards presented lower water absorption and swelling thickness than the ones obtained using NaHCO_3_ in the extraction (GC_E2_).

Although the water-resistance properties were lower than the requirements of EN 312 for general purpose boards for use in humid conditions, the aim of this part of the research work was to produce a new 100% biobased particleboard with applicability in the development of food-packaging solutions. Considering the end application suggested, the possibility of producing a new product 100% biobased (based on a food industry by-product) that retains its structural integrity after 24 h water treatment is a very interesting advance.

This work shows that it is possible to produce a new lignocellulosic material from an underused by-product such as grape cane, using only citric acid as a crosslinking agent.

## 4. Conclusions

The feasibility of the grape canes to be used in material industry production was demonstrated.

This work demonstrates the viability of using grape cane particles as raw material in particleboard production, combined with a melamine formaldehyde urea (MFU) resin catalysed with citric acid. The influence of the grape cane particles, in the original condition and after undergoing extraction, on the particleboards properties was studied, showing the possibility of a global valorisation of all this viticulture by-product. To our knowledge, this was the first time that extracts from grape cane by-product were assessed for its applicability in materials industry production.

The self-condensation reaction of the grape cane extracts and the curing reaction with citric acid were studied, showing the feasibility of this extracts to be used in new bioadhesive formulation. Citric acid was demonstrated to be a good crosslinking agent for grape cane extract. The viability of the bio-adhesive was demonstrated. The produced particleboards do not comply with the requirements of CEN/TS 16368 (Type LP1). Nonetheless, the possibility of using the same industrial by-product as a particle raw material and as a component of the binder is a very interesting route to produce sustainable packaging products, without using toxic or petroleum-based adhesive systems. The resulting bio-based particleboards can be reused, recycled or composted. This work could be the starting point for future development of new food-packaging products with low environmental impact, based on the valorisation of grape cranes.

## Figures and Tables

**Table 1:** GC Extraction conditions on the extraction yield.

Extraction Agent		S/L	Temperature (°C)	Codification
NaOH (%)	NaHCO_3_ (%)			
1	0	1:10	90	CG_E1_
0	2	1:10	90	CG_E2_

**Table 2:** Yield for different extraction conditions.

Nomenclature	Extraction Conditions		Extraction Yield (%)
	NaOH (%)	NaHCO_3_ (%)	
Extract 1	1	0	15.85 ± 3.28
Extract 2	0	2	13.17 ± 2.12

**Table 3:** FTIR peak assignment of grape cane after and before the extraction process.

GC		GG_ER1_		GG_ER2_		Group	Range
cm^−1^	Intensity	cm^−1^	Intensity	cm^−1^	Intensity		
3351	20.7 ± 0.8	3340	19.7 ± 0.9	3346	18.2 ± 0.7	-OH stretch	3336
2916	12.0 ± 0.2	2916	7.6 ± 0.3	2917	8.5 ± 0.1	-CH_2_- asymmetric stretch	2916–2936
2850	10.8 ± 0.4	2853	6.9 ± 0.1	2852	7.3 ± 0.2	-CH_2_- symmetric stretch	2843–2863
1732	24.5 ± 0.1	1730	16.2 ± 0.2	1737	17.4 ± 0.2	C=O stretch in unconjugated ketones, carbonyls and in ester groups (hemicellulose)	1738
1681	11.4 ± 0.6	--	--	--	--	C=O vibration in carboxylic group of phenolic acids (p-hydroxybenzoic acid)	1676
1596	28.6 ± 0.2	1598	24.0 ± 0.2	1595	17.8 ± 0.5	Aromatic skeletal vibration and C=O stretch (lignin)	1595
1509	14.0 ± 0.3	1504	9.4 ± 0.1	1504	10.7 ± 0.1	C_AR_=C_AR_ (Pp cd.)	1500–1600
1455	17.1 ± 0.5	1453	14.0 ± 0.5	1453	15.1 ± 0.3	C=C and C-H bond O-H in plane deformation (lignin and hemicellulose)	1450–1453
1423	19.0 ± 0.2	1425	15.9 ± 0.2	1423	17.1 ± 0.1	CH- deformation; asymmetric in -CH_3_ and -CH_2_- (cellulose)	1430–1485
1373	20.6 ± 0.2	1369	17.2 ± 0.3	1369	17.8 ± 0.2	CH deformation (cellulose and hemicellulose)	1372
1322	15.8 ± 0.1	1320	16.7 ± 0.6	1323	16.1 ± 0.5	Ph-CHR-OH deformation	1260–1350
1239	31.7 ± 0.6	1237	25.4 ± 0.1	1235	25.8 ± 0.2	Syringyl ring and C=C stretch in lignin and xylan	1235
1154	22.4 ± 0.4	1157	21.7 ± 0.3	1156	22.0 ± 0.3	Involves C-O stretching of C-OH/C-O-C (cellulose)	1160
1102	49.7 ± 0.3	1103	49.3 ± 0.1	1103	47.2 ± 0.4	C-O-C stretch (cellulose and hemicellulose)	1105
1072	61.9 ± 0.1	--	--	--	--	C-O primary alcohol stretching (Pp cd)	1060–1070
1051	89.9 ± 0.2	--	--	--	--	C-O-C aromatic ethers symmetric stretch (pyranose ring)	1010–1050
1031	100.0 ± 0.0	1032	100.0 ± 0.0	1031	100.0 ± 0.0	C-O, C-C, and C-C-O stretch (cellulose, hemicellulose, and lignin)	1025–1035
897	7.4 ± 0.9	897	7.7 ± 0.8	897	9.8 ± 0.6	C-O-C aromatic ethers, symmetric stretch	810–850

Ph: Phenyl group; GC_ER1_: GC Extraction residue process1, GC_ER2_: GC Extraction residue process 2_,_ GC_E1_: Extract process 1; GC_E2_: Extract process 2.

**Table 4:** FTIR peak assignment of grape cane extracts GG_E1_ and GG_E2_.

GG_E1_		GG_E2_		Group	Range
cm^−1^	Intensity	cm^−1^	Intensity		
3418	37.1 ± 0.8	3418	32.5 ± 1.1	-OH stretch	3336
2920	21.4 ± 0.2	2920	17.5 ± 0.3	-CH_2_- asymmetric stretch	2916–2936
2851	16.6 ± 0.1	2851	14.0 ± 0.2	-CH_2_- symmetric stretch	2843–2863
1728	15.7 ± 0.1	1739	10.2 ± 0.2	C=O stretch in unconjugated ketones, carbonyls and in ester groups	1738
1681	31.7 ± 0.4	1681	27.7 ± 0.2	C=O vibration in carboxylic group of phenolic acids (p-Hydroxybenzoic acid)	1676
1593	100.0 ± 0.0	1591	100.0 ± 0.0	C_AR_=C_AR_ (Pp)	1500–1600
1462	18.6 ± 0.1	1461	14.4 ± 0.2	C_AR_= C_AR_ (Benzene skeleton) (SB) [26]	1460–1465
1454	24.0 ± 0.3	1454	18.0 ± 0.1	C-H bending of CH_2_ groups (Pp cd.)	1450–1455
1402	58.8 ± 0.2	1402	68.2 ± 0.3	C-C stretching vibration (SB) [26]	1380–1400
1325	34.6 ± 0.1	1326	36.1 ± 0.1	Ph-CHR-OH deformation	1260–1350
1259	25.7 ± 0.1	1259	23.9 ± 0.2		
1204	14.1 ± 0.1	--	--	Ph-OH stretching vibration	1180–1260
1146	46.6 ± 0.3	1145	37.8 ± 0.4	C_AR_-OH linkage due to the presence of oligomer components (SB) [26]	1155
1076	89.2 ± 0.2	1074	96.1 ± 0.5	C-O primary alcohol stretching (Pp cd)	1060–1070
1020	129.3 ± 0.1	1019	100.0 ± 0.2	Aromatic cycle bending (Pp cd) [27]	1025
952	24.6 ± 0.2	953	26.4 ± 0.1	Bending vibration of C=C-H (SB) [28]	965–988
796	23.8 ± 0.3	--	--	C-O-C aromatic ethers, symmetric stretching	810–850
762	29.2 ± 0.6	760	26.9 ± 0.3	C-C Alkanes skeletal vibrations	720–750

Ph: Phenyl group; GC_E1:_ Extract process 1; GC_E2:_ Extract process 2; Pp cd. Condensed tannins, SB: Stilbenes.

**Table 5:** FTIR peak assignment of grape cane extracts cured alone and with citric acid at 120 °C.

GG_EC1_		GG_E1CAC_		Group	Range
cm^−1^	Intensity	cm^−1^	Intensity		
3409	26.2 ± 0.2	3426	22.4 ± 0.1	-OH stretch	3300–3400
3391	26.1 ± 0.3	3266	9.3 ± 0.1		
2920	12.8 ± 0.1	2921	9.1 ± 0.1	-CH_2_- asymmetric stretch	2916–2936
2851	10.8 ± 0.1	2851	5.9 ± 0.2	-CH_2_- symmetric stretch	2843–2863
1767	7.6 ± 0.0	--	--	C=O stretch in unconjugated ketones, carbonyls and in ester groups	1738
--	--	1715	126.7 ± 0.3	Axial stretching of carbonyl groups (C=O) in carboxylic acid group (citric acid)	1700–1750
1591	100.0 ± 0.0	1598	100.0 ± 0.0	C_AR_=C_AR_ (Pp.)	1500–1600
1395	55.3 ± 0.1	1394	70.1 ± 0.4	Ph-CHR-OH deformation	1260–1350
1262	25.6 ± 0.2	--	--		
--	--	1200	71.6 ± 0.6	C-O bond of -O-(C=O)- stretch [14,29]	1215
1074	85.7 ± 0.1	1075	69.3 ± 0.2	C-O primary alcohol stretching (Pp)	1060–1070
1020	115.7 ± 0.3	1023	90.5 ± 0.6	Aromatic cycle bending (Pp)	1025
--	--	890	15.6 ± 0.3	C-O-C aromatic ethers, symmetric stretch	810–850
760	18.3 ± 0.1	796	14.2 ± 0.2	C-C Alkanes skeletal vibrations	720–750

Ph: Phenyl group; Pp: Polyphenols; GC_EC1_: Extract 1 cured at 120 °C; GC_E1CAC_: Extract 1 with citric acid cured at 120 °C.

**Table 6:** Polymerization degree for the GC_E1_ + CA (33% on adhesive weight, dry basis) cured at 100 –180 °C during 120 s.

Wavenumber (cm^−1^)	Type of Bond	Curing Temperature	Band Area
3000–3300	-OH stretching	100 °C	68.8 ± 1.4
		120 °C	63.2 ± 0.9
		140 °C	35.7 ± 0.3
		160 °C	15.4 ± 0.6
		180 °C	15.6 ± 0.8
3300–3700	-OH stretching	100 °C	141.5 ± 1.6
		120 °C	135.2 ± 2.0
		140 °C	101.7 ± 1.3
		160 °C	51.2 ± 0.8
		180 °C	53.6 ± 1.5
2800–3000	-CH_2_- stretching	100 °C	14.8 ± 0.1
		120 °C	16.9 ± 0.2
		140 °C	21.9 ± 0.1
		160 °C	27.8 ± 0.3
		180 °C	27.7 ± 0.1
1670–1780	C=O stretching	100 °C	33.0 ± 0.2
		120 °C	33.3 ± 0.1
		140 °C	41.6 ± 0.3
		160 °C	44.8 ± 0.8
		180 °C	57.3 ± 0.1
1400–1500	C-C deformation	100 °C	64.1 ± 0.2
		120 °C	63.3 ± 0.5
		140 °C	62.9 ± 0.1
		160 °C	57.1 ± 0.6
		180 °C	60.4 ± 0.4
1170–1280	C-O stretching	100 °C	34.3 ± 0.1
		120 °C	32.6 ± 0.2
		140 °C	31.5 ± 0.1
		160 °C	20.1 ± 0.9
		180 °C	17.9 ± 1.1

GC_E1_: Extract process 1; CA: Citric Acid.

**Table 7:** Polymerization degree for the GC_E1_ + CA (43% on adhesive weight, dry basis), cured at 100–160 °C during 120 s.

Wavenumber (cm^−1^)	Type of Bond	Curing Temperature	Band Area
3000–3300	-OH stretching	100 °C	52.9 ± 2.1
		120 °C	29.3 ± 0.9
		140 °C	9.1 ± 0.1
		160 °C	7.9 ± 0.2
3300–3700	-OH stretching	100 °C	128.8 ± 0.6
		120 °C	100.6 ± 1.3
		140 °C	54.3 ± 0.9
		160 °C	43.2 ± 0.2
2800–3000	-CH_2_- stretching	100 °C	34.2 ± 0.2
		120 °C	35.9 ± 0.1
		140 °C	35.8 ± 0.3
		160 °C	36.4 ± 0.2
1670–1780	C=O stretching	100 °C	153.3 ± 0.2
		120 °C	179.2 ± 0.1
		140 °C	182.9 ± 0.3
		160 °C	186.7 ± 0.6

GC_E1_: Extract process 1; CA: Citric Acid.

**Table 8:** Results of particleboards bonded with MFU resin and with recycled wood.

Type of By-Product	Density (kg/m^3^)	Dry IB Strength (MPa)	24 h in Water-Thickness Swelling (%)
IW	686 ± 2	0.59 ± 0.02	24.9 ± 0.8
GC	616 ± 9	0.62 ± 0.04	16.9 ± 0.8
GC_ER1_	606 ± 2	0.65 ± 0.01	23.3 ± 2.1
EN 312 Requirement (type P2)		≥0.35	--

Values are presented as mean ±standard deviation (*n* = 6); IB = Internal Bond; GC = Grape cane; GC_ER1_ = Grape cane extraction residue; IW = Industrial mix wood particles; Particleboard dimensions 210 × 210 × 16 mm.

**Table 9:** Results for grape cane particleboards bonded with GC_E_ and citric acid.

Type of Adhesive	Material Used as Particles	Density (kg/m^3^)	IB Strength (MPa)	Bending Strength (N/mm^2^)
(67%) GC_E1_ + (33%) CA	GC	534 ± 9.2	0.16 ± 0.01	3.26 ± 0.30
(67%) GC_E1_ + (33%) CA	GC_ER1_	518 ± 17	0.19 ± 0.02	2.18 ± 0.42
(67%) GC_E2_ + (33%) CA	GC	524 ± 1.4	0.18 ± 0.02	3.18 ± 0.16
(67%) GC_E2_ + (33%) CA	GC_ER2_	526 ± 12	0.16 ± 0.01	2.15 ± 0.20
CEN/TS 16368			0.28	4.0

Values are presented as mean ±standard deviation (*n* = 6); GC_E1_: Grape cane extract obtained with NaOH, GC_E2_: Grape cane extract obtained with NaHCO_3_; GC_ER1_: Solid fraction obtained after NaOH extraction; GC_ER2_: Solid fraction obtained after NaHCO_3_ extraction CA: Citric Acid; IB = Internal Bond.

**Table 10:** Results for grape cane particleboards bonded with GC_E_ and citric acid.

Type of Adhesive	By-Product Used as Particles	Thickness Swelling (2 h in Water) (%)	Water Absorption (2 h in Water) (%)	Thickness Swelling (24 h in Water) (%)	Water Absorption (24 h in Water) (%)	Thickness Swelling (1 Week Dry) (%)
(67%) GC_E1_ + (33%) CA	GC	31.6 ± 1.4	98.6 ± 12.4	39.7 ± 1.0	106.5 ± 1.3	19.2 ± 1.9
(67%) GC_E1_ + (33%) CA	GC_ER1_	46.3 ± 2.3	127.9 ± 2.90	48.9 ± 4.5	145.2 ± 2.1	33.4 ± 9.3
(67%) GC_E2_ + (33%) CA	GC	51.6 ± 0.8	99.0 ± 13.9	55.5 ± 1.8	127.6 ± 6.5	34.2 ± 1.2
(67%) GC_E2_ + (33%) CA	GC_ER2_	49.7 ± 4.3	146.1 ± 11.3	57.3 ± 3.0	147.0 ± 1.4	36.3 ± 9.8

Values are presented as mean ± standard deviation (*n* = 6); GC_E1_: Grape cane extract obtained with NaOH, GC_E2_: Grape cane extract obtained with NaHCO_3_; GC_ER1_: Solid fraction obtained after NaOH extraction; GC_ER2_: Solid fraction obtained after NaHCO_3_ extraction CA: Citric Acid; IB = Internal Bond.

## Data Availability

Data are contained within the article.
